# The role of the backbone torsion in protein folding

**DOI:** 10.1186/s13062-016-0166-x

**Published:** 2016-12-01

**Authors:** Irina Sorokina, Arcady Mushegian

**Affiliations:** McLean, Virginia USA

## Abstract

**Background:**

The set of forces and sequence of events that govern the transition from an unfolded polypeptide chain to a functional protein with correct spatial structure remain incompletely known, despite the importance of the problem and decades of theory development, computer simulations, and laboratory experiments. Information about the correctly folded state of most proteins is likely to be present in their sequences, and yet many proteins fail to attain native structure after overexpression in a non-native environment or upon experimental denaturation and refolding.

**Presentation of the hypothesis:**

We hypothesize that correct protein folding in vivo is an active, energy-dependent process that most likely applies torque force co-translationally to all proteins and possibly also post-translationally to many proteins in every cell. When a site on an unfolded polypeptide is rotationally constrained, torsion applied at another site would induce twisting of the main chain, which would initiate the formation of a local secondary structure, such as an alpha-helical turn or a beta-turn/beta-hairpin. The nucleation of structural elements is a rate-limiting, energetically unfavorable step in the process of protein folding, and energy-dependent chain torsion is likely to help overcome this barrier in vivo. Several molecular machines in a cell, primarily ribosomes, but also possibly signal recognition particles and chaperone systems, may play a role in applying torque to an unfolded protein chain, using the energy of GTP or ATP hydrolysis. Lack of such force in the in vitro systems may be the main reason of the failure of many longer proteins to attain the correct functional conformation.

**Testing the hypothesis:**

The hypothesis can be tested using single-molecule approaches, by measuring directly the forces applied to polypeptide chains under controlled conditions in vitro, and in bulk, by assessing folding rates and extent of misfolding in proteins that are engineered to experience transient spatial constraint during their synthesis.

**Implications of the hypothesis:**

Learning about the role of main chain torsion in protein folding will improve our understanding of folding mechanisms and may lead to bioengineering solutions that would enhance the yields of correctly folded proteins in heterologous expression systems.

**Reviewers:**

This article was reviewed by Frank Eisenhaber, Igor Berezovsky and Michael Gromiha.

## Background

Protein folding is a multifaceted problem that is being addressed through the examination of two groups of questions. First, there are thermodynamic questions of how the native spatial structure of a protein, complete with the set of the interatomic interactions stabilizing it, is encoded by and can be predicted from the amino acid sequence. Second, there are kinetic questions concerning the set of forces, pathways and folding intermediates that enable proteins to fold correctly and quickly under experimental conditions [1]. The fundamental assumption, ever since the Anfinsen’s classical work [2–4], is that the complete set of instructions for correct protein folding is contained in its sequence, and therefore that studies of isolated protein molecules should provide most of the answer to these questions.

The protein folding processes in a living cell, however, take place in the presence of partially synthesized protein intermediates, co-factors and interacting molecules, in a highly crowded medium that includes protein-targeting molecular machines such as ribosomes, translocons, chaperones, etc., as well as post-translational processing and quality-control machinery. This is quite different from the conditions found even in the most complex in vitro systems, so we may expect that protein folding in a cell proceeds in ways not always accounted for by in vitro studies.

We hypothesize a force contributing to the speed and/or efficiency of protein folding to its native conformation in vivo. This force is torsion that may be applied to a protein main chain by various molecular machines in the cell that are powered by ATP, GTP, or other energy sources.

## Presentation of the hypothesis

A full theory that relates protein primary structure to the speed and accuracy of its folding is still not available. The hypothesis of hierarchical protein folding, which posits that the major elements of secondary structure form independently and assemble into a mature fold, has been examined over the years, but has given way to other explanations, which appear to be better compatible with the experimental evidence. One class of current theories of folding relies on the existence of a molten-globule transition state, where a high fraction of amino acid residues is found within helices and strands, and at the same time there is no tightly packed interior core [5]. A contrasting “zipping-and-assembly” model of protein folding does not demand a molten-globule intermediate [6], but suggests that small elements of secondary structure, such as beta-hairpins and alpha-helical turns, form at many independent sites along the chain and then grow by extension (zip) or coalesce (assemble) with other such elements.

Despite the differences between these and other views on protein folding mechanisms, all of them require some extent of secondary structure to emerge early in the process of formation of the native protein structure, typically at a local scale (see also [7, 8]). Studies examining the folding of short peptides, where distinct steps of structure formation could be monitored directly, suggested that the initiation (“nucleation”) of the first alpha-helical turn is the rate-limiting step in the formation of an alpha-helix (on average, it takes about as much time to form the first turn of a helix as all the other turns combined). Studies of beta-sheet stability have similarly indicated that the formation of beta-turns and beta-hairpins is likely to be the rate-limiting step in sheet assembly [5].

From a physical point of view, these turns, twists and hairpins are precisely the types of structural elements that would be induced if a torque force was applied to the longitudinal axis of a long thin cylinder between two points that were fully or partially restricted from rotating. In fact, just one distal point has to be fixed, if the torque is applied at another point that is itself stabilized in space. Torsion of a linear biopolymer is a notion familiar to molecular biologists from studies of the topology of the DNA double helix. It is well known that if the ends of a double-stranded DNA molecule are covalently linked to each other, or if they are restricted in mobility by interaction with other molecules, then the torque applied to the main chain of the molecule will result in negative or positive supercoiling [9]. Sometimes omitted from this account is a more general rule, i.e., that a twist of any string, such as a single-strand linear polymer, will also induce secondary structure. This has been studied more recently with single-stranded DNA [10], and there is no reason why similar forces applied to a polypeptide should not produce qualitatively similar outcomes, i.e., turns and twists of the molecule.

A quantitative physical model of protein torsion and twist, which would take into account the geometry of the chemical bonds and energetically favorable conformations within the protein main chain, the effects of chain elasticity and viscosity of the solution, as well as the molecular interactions of the side chains and the solvent molecules, is beyond the scope of this paper. Here, we would like instead to discuss the intracellular structures and processes that could result in the application of torque to a polypeptide. In order for chain twisting to be a significant component of protein folding *in vivo*, those twisting forces should be available during the maturation of many classes of proteins, and the process has to be supplied with external energy. Many molecular machines may interact with unfolded, partially folded or misfolded proteins in the cell in an energy-dependent manner, releasing proteins with native three-dimensional structure. These include signal recognition particles, secretion systems, chaperone systems, and protein processing modules. We think, however, that an even more universal device spends energy specifically to introduce a twist of the nascent peptide chain and thereby facilitates their subsequent folding: the ribosome itself.

Stereochemical modeling on partially solved structures of the ribosomal large subunit have predicted 30 years ago that helical twisting of the nascent peptide occurs in the ribosomal exit tunnel [11]. Recent experimental data confirm that a partially helical conformation is attained by certain peptides in the exit tunnel and exit vestibule [12–14].

A rough estimate of energy balance during the ribosomal cycle suggests that the hydrolysis of two GTP molecules and deacylation of an aminoacyl-tRNA bond liberates ~30 kcal/mol of amino acid, only a small fraction of which is consumed for positioning of the incoming charged tRNA that facilitates the peptide bond formation, whereas the rest is thought to dissipate as heat ([15], p. 159). Even if we consider other energy expenditures, such as tRNA translocation or motions of ribosome parts, the energetic needs of twisting the protein chain might be more than covered by the energy surplus in a ribosomal cycle. On the other hand, formation of an alpha helix of the length typical of globular proteins, or of a beta hairpin, may require overcoming a barrier of about only 1-5 kcal/mol of amino acid (calculated based on the data from [16]).

The structural elements of the ribosome that would be able to relay some of this energy into a torque force on the chain are not known. Another crucial question concerns the locations at which the torsion could be applied, though the peptidyl transferase center itself may be a possibility. As for the mechanisms of constraining the torsional mobility of the chain at a downstream site, it is plausible that any of the systems that are able to bind a nascent protein chain, including the aforementioned modules of maturation, sorting, and reactivation of specific subsets of proteins, can play this essential role. In addition, some of those molecular machines may be able to apply their own rotational force to their specific substrates. Cooperation between the ribosome and other machines may be particularly important for the formation of beta-hairpins, which typically bear a twist not fully explained by the current theories and may be too large to fit into the exit vestibule of the ribosome [17] (compare, however, with results in [14]).

## Testing and implications of the hypothesis

Several directions of further work may provide supporting evidence for the hypothesis presented here. First, the plausibility of a co-translational torque applied to the growing peptide chains by the ribosome should be investigated by stereochemical modeling, using the increasingly accurate knowledge of the functional morphology of the translating ribosome. Detailed energy calculations of the transition between relaxed and twisted conformations should also be possible.

Independently of modeling approaches, single-molecule techniques may be employed to measure polypeptide backbone rotation-induced tension upon either folding or unfolding, and also to apply controlled amounts of torsion to defined sites on an unfolded polypeptide and monitor the emergence of local structure directly. Single-ribosome studies have been applied to study behavior of individual molecules in the ribosome cycle, including analysis of pulling forces that are applied to the nascent peptide [18–20]. Forces acting on peptides inside the working chambers of other molecular machines are also being examined [21]. Thus, the experiments to assess torque on the main chain of a nascent peptide are within the realm of possibility. As a side note, nanotechnology applications of experimental protein twisting can also be envisaged, to produce higher-order polypeptide structures that may be not known in vivo but have interesting topology or practical utility.

In addition to approaches that measure the twist and torque of a single protein chain directly, various indirect tests could be also conducted in vitro and in vivo. One in vivo approach could be to express proteins under treatments that may affect the ability of the protein chains to twist co-translationally and post-translationally. For example, synthetic biology may be employed to introduce affinity tags in various parts of an expressed protein, and the ligands for these tags may be exposed in cells either in a soluble or immobilized form, to see whether a transient restriction of rotational mobility in a growing chain produces a higher proportion of the native form of the protein. As our understanding of the torsional effects on protein folding improves, we may devise new ways to improve the yields of native protein in the context of industrial production, and develop protocols for refolding denatured proteins by applying controlled torque to them.

In conclusion, we hypothesize that energy-dependent twisting of a protein main chain, applied co-translationally by the ribosome itself and possibly post-translationally by other molecular machines, may be an overlooked factor that affects protein folding in general and becomes critical for efficient folding of longer proteins.

## Reviewers’ comments

### Reviewer’s report 1: Frank Eisenhaber, Bioinformatics Institute, A*Star, Singapore

## Reviewer comments

The authors hypothesize that the protein folding process itself might need active, energy-spending support to overcome conformational barriers, especially at the level of nucleation of secondary structural elements. They suggest to verify the idea by measuring the torque on chain torsion in single-molecule experiments. The thoughts are interesting and provoking.

Authors’ response: *We are grateful to Dr. Eisenhaber for his positive opinion of our work.*

## Reviewers’ comments

### Reviewer’s report 2: Igor Berezovsky, Bioinformatics Institute, A*Star, Singapore

## Reviewer comments

The most appealing aspect in presented hypothesis, which is discussed since Levinthal’s original work in 1968, is co-translational nature of the protein folding process. It is satisfactory to see that many recent works on protein folding turned in the direction of the co-translational process in crowded cellular environment. This hypothesis is another contribution that supports correct formulation of the protein folding problem. The role of backbone torsion as the driving force of the folding process fueled by the energy ATP/GTP and provided by cellular machinery is of interest, but requires further justification to be proposed and more explanations on how it can be tested in experiment and quantified in theoretical models. Specifically, it would be important to discuss why backbone torsion is the origin of the folding and not the consequence of the combination of different forces that work in the protein structure.

Authors’ response: *We are grateful to Dr. Berezovsky for his interest in our hypothesis and agree that the next steps are to develop theoretical models of backbone torsion. We note that our hypothesis does not postulate that the backbone torsion is the sole origin or sole driving force of folding. In fact, many proteins are folded into the correct conformation in vitro in the absence of any cellular machines. On the other hand, if indeed applied by the ribosome or other energy-consuming machines, backbone torsion would be a ubiquitous initial early folding factor. Multiple other factors contribute to the folding process and finely tune it after the initial twist of the backbone occurs. Some reverse “untwisting” or relaxing must occur in the later folding stages too, since we do not observe uniform twisting of the backbone in all correctly folded proteins.*

## Reviewer comments (continued)

Why energy should be spend to apply torques, instead of opportunistic use of attraction/repulsion abundant in the crowded environment?

Authors’ response: *Our main postulate is that many proteins, in particular larger ones, may not realistically achieve their native folded state without the proposed energy-dependent twisting step. This is strongly suggested by the multitude of empirical observations of the failure of many long proteins to fold into correct conformation in vitro, i.e. in the absence of the postulated twist-inducing cellular machinery. Attraction and repulsion between atoms of protein and the solvent remain in effect, of course, and come into play to stabilize the twisted chain. As for the expense of energy, in the specific case of the ribosome, it appears that the excess energy is there to be spent (see text).*

## Reviewer comments (continued)

It would be useful to estimate the energy cost of other interactions, such as electrostatics, van der Waals, hydrogen bonds, necessary to achieve required backbone torsion, in order to claim the important role of torsion hypothesized here.

Authors’ response: *We agree. Our work in progress concerns the estimation of the forces and energy expenditures that are required to induce the chain torsion in the first place and, equally important, to stabilize and maintain the twist along the protein backbone.*

## Reviewer comments (continued)

Finally, it does not seems trivial to design an experiment, which would allow one to survey the twisting of the polypeptide chain during the folding process and to single out the contributions of torsion and other interactions. It would be interesting to learn about authors’ ideas on the design and implementations of such experiment.

Authors’ response: *We have described briefly some such experiments in the “Testing and implications of the hypothesis” section.*

## Reviewers’ comments

### Reviewer’s report 3: Michael Gromiha, Indian Institute of Technology Madras

## Reviewer comments

In this manuscript the authors hypothesized that torsion is an important factor, which initiate the formation of secondary structure and folded native state. It may be acceptable in a way. However, no attempt has been made to prove the hypothesis.

Authors’ response: *We thank Dr. Gromiha for his comments. This study was submitted under the “Hypothesis” category. Proving it will most likely require a community effort. The ultimate proof will be the successful application of controlled twisting force to achieve correct folding in vitro for those overexpressed and denatured proteins that have so far, in the course of multiple refolding experiments in various in vitro conditions, evaded all attempts to refold them in native conformations.*

## Reviewer comments (continued)

1. Folding initiating residues are known for several proteins. This should be checked. 2. Folding rates are known for several proteins. The concept may be tested with slow and fast folding proteins. 3. Easy and difficult samples in CASP experiment could be tested.

Authors’ response: *1. Our hypothesis of backbone twisting does not contradict the existence of the folding-initiating residues; chain twisting is another, independent factor in the process of protein folding. 2. Our hypothesis currently does not address the specific values of folding rates in different proteins. Future modeling should be able to address this. 3. The most difficult CASP targets, i.e., those that have no templates with known structure, might indeed become easier to predict if chain twist proves to be a factor in the protein folding process and is incorporated into prediction algorithms.*
